# Impact of feeding diets without mineral P supplement on the immune system of two laying hen strains

**DOI:** 10.1016/j.psj.2026.106771

**Published:** 2026-03-13

**Authors:** Nadine Wallauch, Sonja Schmucker, Tanja Hofmann, Vera Sommerfeld, Martin Hasselmann, Korinna Huber, Markus Rodehutscord, Volker Stefanski

**Affiliations:** Institute of Animal Science, University of Hohenheim, Stuttgart 70599, Germany

**Keywords:** Laying hen, Immune system, Phosphorus renunciation, Leukocyte population, Cytokine expression

## Abstract

Minimizing the addition of mineral phosphorus (P) to diets of laying hens is of great interest in poultry farming and might be possible due to the ability of poultry to utilize phytate. However, certain changes in nutrient composition can impair the immune system and affect animal health and welfare. The objective of this study was to investigate the impact of a complete renunciation of mineral P supplementation on the immune system of 2 commercial laying hen strains, before and after the onset of egg laying. At the age of 15 and 20 wk, Lohmann Brown-Classic (LB) and Lohmann LSL-Classic (LSL) hens were placed in metabolic units and fed a corn-soybean-based diet either without supplemented P (P-) or with 1 g/kg supplemented mineral P (P+). After 4 wk, numbers of various leukocyte types in blood, spleen and cecal tonsils, functionality of lymphocytes and gene expression of pro- and anti-inflammatory cytokines were measured. The P- diet increased proportions of natural killer cells and CD4^+^CD25^high^ T cells in the cecal tonsils. Moreover, expression levels of IL-2 and IL-10 at the age of 24 wk were higher with the P- diet compared to the P+ diet. However, the P- diet led to lower immune cell counts in the blood, such as total leukocytes, total T cells and CD4^+^ T cells. No effects of mineral P renunciation were seen in blood antibody concentrations and splenocyte proliferation. Likewise, immune cell counts in spleen and cecal tonsils were not affected. Our findings suggest that the renunciation of mineral P supplementation modifies immune regulation within the gut-associated immune system and fosters an anti-inflammatory condition. In addition, higher immune cell subpopulations related to innate and humoral immunity as well as higher IFN-γ and SOD2 expression in general were found in LB hens, whereas a more pronounced cellular immunity and higher expressions of IL-1β, IL-10, TNF-α and iNOS were found in LSL hens, reconfirming and extending our knowledge of strain-specific differences.

## Introduction

Phosphorus (P) is an essential nutrient for several biological processes, an adequate P availability is also critical for a functional and well-regulated immune system (Taylor and Bushinsky, 2009; Heyer et al., 2015; Bryden et al., 2021). In plant seeds, the main component of poultry feed, organic P is mainly bound as phytic acid (InsP_6_) or its salts (phytate), which were thought to be hardly metabolized by poultry (Selle et al., 2009; Jing et al., 2018). Chicken feed is therefore often selectively supplemented with mineral P (Soares, 1995; Smil, 2000; Selle et al., 2009). In terms of sustainable animal husbandry, reducing mineral phosphorus (P) is becoming increasingly important, particularly in the world's largest livestock farming sector, poultry production. Contrary to initial assumptions, an increasing number of studies suggest that laying hens are capable of utilizing phytate-bound P to some extent, provided that the ration contains little amounts of mineral P (Jing et al., 2018; Sommerfeld et al., 2020a,b; Rodehutscord et al., 2023). Recent studies indicated that reduction of mineral P increases the endogenous phytase activity in the intestine, which enables the degradation of InsP_6_ to lower *myo*-inositol phosphates (InsP_x_) and *myo*-inositol (MI), thus enhancing P release and intestinal phytate-P uptake (Abudabos, 2012; Zeller et al., 2015; Sommerfeld et al., 2018, 2024).

Despite the recommendation to reduce mineral P in laying hen husbandry (Rodehutscord et al., 2023), there are scarcely any studies dealing with mineral P reduction, especially with regard to the immune system. In general, increasing available P is suggested to have beneficial effects on the adaptive immune system in several species, as reviewed by Heyer et al. (2015). Nevertheless, it is assumed that the amount of mineral P in laying hen feed can be significantly reduced without negative consequences for the immunocompetence of the animals (Li et al., 2017; Hofmann et al., 2021). Our group recently demonstrated that a moderate reduction of dietary mineral P by 20 % does not negatively impact the immune system of high-performing laying hens. On the contrary, the observed results even suggested positive effects on their immune system, such as increasing numbers of innate and adaptive immune cells and higher antibody concentrations (Hofmann et al., 2021). We assumed that MI and InsP_x_, for which immunomodulatory capacities have already been described (Feske, 2007; Miller et al., 2008; Sauer and Cooke, 2010), could be involved in mechanisms by which diets fostering the degradation of phytate affect the immune system. Taken together, degradation products of InsP_6_, especially MI, seem to impinge on inflammatory pathways and to contribute to a low-inflammatory immune regulation. Such effects have already been shown in mammals (Piranlioglu et al., 2019; Bizzarri et al., 2020), but were not investigated in poultry so far.

As Hofmann et al. (2021) found immunological effects already at a moderate dietary mineral P reduction, the question arises whether even more pronounced effects will be present when laying hens are forced to increase the endogenous hydrolyzation of phytate by a complete renunciation of mineral P. Thus, the objective of the present study was to compare the impact of a complete renunciation of dietary mineral P with a diet formulated according to most recent research recommendations (Rodehutscord et al., 2023). We analyzed major immune cell types in peripheral and lymphoid tissues and evaluated lymphocyte functionality. Moreover, going beyond our previous study, we focussed on the cytokine expression profile of various pro- and anti-inflammatory cytokines in order to evaluate the inflammatory state of immunologically important tissues in the context of a complete renunciation of mineral P. As previous studies had found pronounced strain-specific differences in the immune system in general and in the immune-modulatory properties of mineral P-reduced diets in particular (Habig et al., 2012; Hofmann et al., 2021), 2 different high-performance laying hen strains were used herein. In addition, two distinct production phases were evaluated – before and after the onset of egg laying – as previous studies have demonstrated stage-dependent effects on physiological parameters related to P metabolism and immune competence (Sommerfeld et al., 2020a, Schmucker et al., 2021).

## Materials and methods

The present study was part of the interdisciplinary Research Unit P-FOWL: “Inositol phosphates and myo-inositol in the domestic fowl: Exploring the interface of genetics, physiology, microbiome, and nutrition” at Hohenheim university (https://p-fowl.uni-hohenheim.de/). The study was conducted in accordance with German animal welfare legislation and was approved by the local animal ethics committee (Regional Council Tübingen, Germany (Project no. HOH67-21TE)).

The experimental design has been described in detail by Sommerfeld et al. (2024), which also presents fundamental performance data, precise feed composition and physiological parameters related to feeding. In brief, animals were kept at the Agricultural Experiment Station of the University of Hohenheim, Germany. The 2 × 2 × 2 factorial experiment included the factors strain (Lohman Brown-Classic (LB) and Lohmann LSL-Classic (LSL) (representing 2 well-characterized and established laying hen strains, with a stable and comparable laying performance), production period (before the onset of egg laying (19 wk of age) and after the onset of egg laying (24 wk of age) and dietary P concentration (without mineral P supplementation (P-) or with 1g/kg supplemented P (P+) from monocalcium phosphate). The P+ diet, in this context, represents a diet with a mineral P content that is already moderately reduced compared to official recommendations, and corresponds to the mineral P-reduced diet from the previous study by (Hofmann et al., 2021).

Experimental diets were based on corn and soybean meal without the inclusion of exogenous phytase. They were calculated to contain adequate levels of all nutrients according to the recommendations of the German Society of Nutrition Physiology (GfE), except for P. A detailed composition of the experimental diets can be found in supplementary file S3 Table 1, according to Sommerfeld et al. (2024). The hens of the first period received grower/developer (15-16.5 wk), prelayer (16.5-17.5 wk) and layer feed (17.5-19 wk) and the hens of the second period received a layer feed throughout the period (20-24 wk). Diets were either supplemented with 1 g mineral P/kg feed from monocalcium-phosphate or contained no mineral P supplementation. The experimental diets were provided to the hens upon their placement in the metabolic units in wk 15 or 20 until the end of the experiment. For a total of 4 wk, hens received one of the 2 experimental diets (either P- or P+), which were evenly distributed across strains and periods. A total of 80 hens were used, with 10 replicates per strain, diet and period, 20 hens per diet and strain or diet and period and 40 hens per main treatment, in a randomized complete block design. Feed and water were provided ad libitum throughout the trial. Artificial lightning of 9 h of light and 15 h of darkness was provided during the first period (15-19 wk) and then gradually changed to obtain 16 h of light and 8 h of darkness at the end of the second period (20-24 wk). The barn temperature was set to 18-22°C during the periods. For a detailed description of the experimental design, detailed results of composition and feed analysis, and results of production parameters see Sommerfeld et al. (2024).

### Blood and tissue sampling

For immunological analysis, blood and bile samples, the spleen as a major secondary lymphatic organ, and the cecal tonsils (CT) as important lymphatic tissues in the intestine were collected. Blood samples were taken one wk before slaughter - at 18 wk or 23 wk of age, respectively – by puncturing the vena ulnaris in order to exclude any influence of the slaughter process on blood immune cell numbers. Blood was taken within 4 min and on average 1 min 12 s after the hens' removal from the pen, thereby minimizing the effects of an acute stress response to capture and handling on immune cell distribution in the blood. Blood samples were collected in 5 mg/ml EDTA (Sigma Aldrich, St. Louis, MO). For flow cytometric analysis, part of the blood was fixed by adding TransFix® reagent (#TFB-01-1, Cytomark Ltd., Buckingham, UK) according to the manufacturer's instructions. Stabilized blood samples were stored at room temperature until further processing.

Tissue and bile samplings were conducted in wk 19 or 24 on 2 consecutive days, with treatments evenly distributed over the days. Hens were individually stunned with a gas mixture of 35% CO_2_, 35% N_2_, and 30% O_2_ and killed by bleeding. Trunk blood was collected in tubes containing EDTA, centrifuged (10 min, 2500 × *g*), and the plasma was stored at -20°C until further processing for antibody analyses. After slaughter, the spleen was collected under aseptic conditions and stored for transport on ice in ice-cold PBS (Roth, Karlsruhe, Germany) containing 1% Fetal Bovine Serum (FBS) and 50 µg/mL Gentamycin (both Sigma Aldrich, St. Louis, MO). 1 randomly chosen CT was taken and stored for transport on ice in PBS containing 1% FBS and 50 µg/mL gentamycin. Bile was collected by puncturing the gallbladder, stored on ice for transport, and frozen at -20°C until determination of antibody concentration. The second CT was frozen directly in liquid nitrogen. Shock-frozen samples were stored and transported on dry ice and finally frozen at -80°C until further analysis by quantitative reverse transcription PCR (qRT-PCR).

### Sample preparation

For flowcytometric analyses and functional assays, spleen and CT were processed according to Hofmann and Schmucker (2021) to separate immune cells from the lymphoid tissues. The weight of both spleen and CT was determined. The spleen was cut in half under sterile conditions. One half was directly frozen in liquid nitrogen and stored at -80°C until further analysis by qRT-PCR. The remaining spleen was dissociated using a gentleMACS Dissociator and gentleMACS C-Tubes (#130-096-334) (both Miltenyi Biotec, Bergisch Gladbach, Germany). In order to remove cells from connective tissue, the cell suspension was passed through a 40 µm MACS SmartStrainer and subsequently through a 20 µm MACS SmartStrainer (both Miltenyi Biotec, Bergisch Gladbach, Germany). The filtrate was centrifuged (10 min, 300 × *g*, 4°C) and the cell pellet was resuspended in PBS with 1% FBS. The final volume of the spleen lymphocyte suspension was determined and one sample (1 mL) of cell suspension per hen was stored on ice until further processing for flow cytometric analysis of immune cell numbers. For the functional analyses, the splenocytes were further processed by gradient centrifugation as described by Hofmann et al. (2021). In order to separate mononuclear cells, the cell suspension was loaded onto a gradient (1.077 g/mL Biocoll separation solution, Biochrom, Berlin, Germany) and centrifuged (12 min, 600 × *g*, 20°C). The interphase was removed. Mononuclear cells were washed in PBS + 1% FBS and resuspended in RPMI 1640 + 10% FBS. Cell counts were determined using a Z2 Coulter counter (Beckman Coulter, Krefeld, Germany), and isolated mononuclear leukocytes were immediately processed for the lymphocyte proliferation assay. Intraepithelial lymphocytes (IEL) from one randomly selected CT were dissected from the mucosa by shaking the tissue twice for 20 min under slow rotation in Hanks' Balanced Salt Solution (without Mg^2+^ and Ca^2+^) supplemented with 5 mM EDTA, 5% FBS and 1 mM Dithiothreitol (#RO862, Thermo Fisher Scientific, Waltham, MA). The suspension was placed on a 40 µm MACS SmartStrainer. The filtrate containing the IEL was washed in 2 steps before the single cell suspension was centrifuged. The cell pellet was resuspended in PBS containing 1% FBS. The final volume of the IEL suspension was determined and stored on ice until further processing for flow cytometric analysis.

### Flow cytometric analysis

Different leukocyte types of the stabilized whole blood, the single cell suspensions of the spleen and the IEL of the CT were differentiated and counted via flowcytometric analyses. Immune cells were stained with fluorescently labelled antibodies using a no-lysis-no-wash method described in detail by Seliger et al. (2012) and Hofmann and Schmucker (2021). The following chicken-specific antibody-fluorochrome conjugates were used: Anti-CD45-APC (#8270-11, clone LT40), anti-monocyte/macrophage PE (#8420-09, clone Kul01), anti- CD4-PacBlu, - PE or - PE.Cy7 (#8210-26; #8210-09;#8210-17, clone CT-4), anti-CD3-PE (#8200-09, clone CT-3), anti- CD8α-FITC (#8220-02, clone CT-8), anti-Bu-1-FITC (#8395-02, clone AV20) (all SouthernBiotech, Birmingham, AL), anti-CD41/61 PE (#MCA2240PE, clone 11C3; BioRad, Hercules, CA), anti-TCRγδ-PerCP (#NBP1-28275PCP, clone TCR1, Novus Biologicals, Centennial, CO), anti-CD25-Alexa647 (#MCA5925GA, clone AV142 Biorad, Hercules, CA) self-coupled using an Alexa Flour® 647 Antibody Labeling Kit (#A20186 Invitrogen, ThermoFisher Scientific, Waltham, MA, USA), according to the manufacturer's instructions. In order to exclude non-specific binding of anti-CD45 APC and to verify a correct total leukocyte count, a mouse IgM isotype control (#0101-11, clone 11E10, Southern Biotech) was included. Analyses were performed on a BD FACSCanto^TM^ II (BD Bioscience, Heidelberg, Germany) equipped with a 488 nm blue laser, 630 nm red laser and 405 nm violet laser using BD FACSDiva^TM^ Software II (DB Bioscience).

EDTA-stabilized whole blood was pre-diluted 1:50 and subsequently labelled with an appropriate antibody mixture according to Hofmann and Schmucker (2021). For the labelling of single cell suspensions of splenocytes and IEL from CT, the antibody panel of Hofmann and Schmucker (2021) was extended with the following antibodies: Anti-CD3-PE for differentiation between T cells and non-T cells for the subsequent differentiation between cytotoxic T cells (CTL) and natural killer (NK) cells; anti-CD25-Alexa647 for differentiation of potential regulatory T cells (Tregs) as described in Hofmann et al. (2025). In each case, 50 µL of prediluted EDTA whole blood, or 50 µL of the single cell suspensions of splenocytes or IEL of CT, were incubated with 20 µL of the corresponding antibody mixtures, in a total volume of 70 µL for 45 min at room temperature. Single cell suspensions of splenocytes, as well as IELs, were then incubated with SYTOX Blue Dead Cell Stain (#S34857, Invitrogen, Thermo Fisher Scientific, Waltham, MA) for an additional 10 min to exclude dead cells. The stained cells were suspended in 1000 µL (splenocytes) or 400 µL (whole blood and IEL of CT) PBS supplemented with 2% BSA, 0.1% NaN_3_ and 5 mg/mL EDTA and stored at 6°C until analysis. For the flow cytometric determination of the leukocyte subsets, at least 10,000 CD45^+^ events per sample for whole blood and 30,000 CD45^+^ events per sample for splenocytes and IEL were analysed. Specific immune cell types were classified by the combination of surface marker expressions as follows: Total leukocytes (CD45^+^), thrombocytes (CD45^dim^/CD41/61^+^) (blood and spleen only), monocytes (CD45^+^/Kul01^+^) (blood and spleen only), NK cells (CD45^+^/CD3^-^/CD8α^+^) (spleen and CT only), B cells (CD45^+^/Kul01^-^/Bu-1^+^), CD4^+^ T cells (CD45^+^/CD3^+^/ CD4^+^/TCRγδ^-^/CD8α^+^ or CD8α^-^), γδ T cells (CD45^+^/CD3^+^/CD4^-^/TCRγδ^+^/CD8α^+^ or CD8α^-^), CTL (CD45^+^/CD3^+^/ CD4^-^/TCRγδ^-^/CD8α^+^), CD4^+^/CD25^-^ cells, CD4^+^/CD25^dim^ cells, and potential Tregs (CD4^+^/CD25^high^). Heterophils in the blood were identified based on their FSC/SSC characteristics. Detailed gating strategies for each sample type are provided in supplementary file S1. The absolute number of leukocytes per µL blood or tissue was determined using BD Trucount™ tubes (BD Biosciences, Heidelberg, Germany) according to the manufacturer's instructions. Total leukocyte count was combined with determined cell frequencies to calculate the absolute cell counts of each leukocyte subset.

### Splenic lymphocyte proliferation assay

A lymphocyte proliferation assay was used to examine the mitogen-induced proliferative capacity of splenocytes in vitro as described in Hofmann et al. (2021). Per animal, 1.5 × 10^5^ cells were transferred into a 96-well round bottom cell culture plate (Neolab, Heidelberg, Germany) and were stimulated for 44 h with either 10 µg/mL concanavalin A (ConA), 10 µg/mL poke weed mitogen (PWM) (both Sigma Aldrich, St. Louis, MO), or were incubated without stimulation as a negative control at 41°C and 5% CO_2_. Each treatment was performed in triplicate. After 44 h of incubation, 0.25 µCi ^3^H-thymidine (PerkinElmer, Rodgau, Germany) was added to each well and cells were further incubated for 24 h. Cells were harvested on glass fiber filters (Skatron, Lier, Norway). The amount of incorporated radioactivity was evaluated using a liquid scintillation analyzer (PerkinElmer, Rodgau, Germany). For each triplicate, the mean of cpm was calculated, and delta cpm for ConA- and PWM-stimulated splenocytes was generated (= mean cpm of stimulated cells - mean cpm of non-stimulated cells). The inter-assay CV for delta cpm of ConA was 23.6% and for delta cpm of PWM was 16.9%. The particular well plate was included in the statistical analysis of the splenocyte proliferation data in order to account for the inter-assay variance.

### Enzyme-linked immunosorbent assay

ELISA was used to determine the concentrations of IgM and IgY antibodies in trunk blood plasma, as well as IgA in plasma and bile as described by Hofmann et al. (2021). As capture antibodies, either 200 ng/well of goat-anti-chicken IgA Fc antibody (#A30-103-A), goat-anti-chicken IgM antibody (#A30-102-A) or 100 ng/well goat-anti-chicken IgY antibody (#A30-104-A) (all Bethyl Laboratories, Montgomery, TX) were used. Antibodies were coated on 96-well flat bottom microtiter plates (Thermo Fisher Scientific, Waltham, MA) and incubated over night at 4°C. Plates were blocked with BSA (Roth, Karlsruhe, Germany) for 30 min and dilutions of plasma (IgA: 1:2,000; IgM: 1:10,000; IgY: 1:400,000) and bile samples (IgA: 1:1,000,000) were added in triplicates and incubated for 1 h at room temperature. In order to detect captured antibodies, either 2 ng/well of horseradish peroxidase-labelled goat anti-chicken IgA Fc (#A30-103-P) or 1 ng/well goat anti-chicken IgM (#A30-102-P) or goat anti-chicken IgY (#A30-104-P) (all Bethyl Laboratories, Montgomery, TX) were used, respectively. Absorbance was measured at 450 nm. Antibody concentrations were quantified by reference to a calibration curve set up with pooled plasma controls based on known concentrations of chicken IgA, IgM and IgY. The Ig concentrations of the reference sample was previously determined using chicken IgG ELISA Kit (#E33-104), chicken IgM ELISA Kit (#E33-102) and chicken IgA ELISA Kit (#E33-103) (all Bethyl Laboratories, Montgomery, TX). CV of intra- and inter-assay was 3.1% and 7.2% for IgA, 3.8% and 9.4% for IgM, 6.7% and 7.7% for IgY in plasma, and 4.3% and 2.4% for IgA in bile.

### Gene expression analysis by quantitative reverse transcription PCR

For gene expression analysis, RNA of spleen and CT were extracted using Trizol Reagent (#15596018, Invitrogen, Thermo Fisher Scientific, Waltham, MA) according to the manufacturers’ protocol. RNA was extracted from 100 mg tissue. Samples were homogenized using 2.8 mm stainless steel beads at 5.5 m/s for 40 s on a FastPrep^TM^ FP120 (Thermo Electron Corporation, Waltham, MA). Samples were incubated for 5 min (for spleen) or 10 min (for CT). A centrifugation step, as recommended for samples with high fat content, was included afterwards. Isopropanol-precipitated RNA was dissolved in nuclease-free water. Concentration and quality of the RNA were measured using a NanoDrop2000 Spectrophotometer (Thermo Fisher Scientific, Waltham, MA), provided in supplementary file S2. In addition, the integrity of extracted RNA of a random but representative subset (including all tissues and treatments) was checked on a Qubit 4 using the Qubit RNA IQ Assay Kit (#Q33221) (both Thermo Fisher Scientific, Waltham, MA) and via gel-electrophoresis (Aranda et al., 2012). Samples were stored at – 80°C until further processing.

In total, the genes of ten cytokines (IL-1β, IL-2, IL-4, IL-6, IL-10, IL-12, IL-13, IL-17, tumor necrosis factor alpha (TNF-α), interferon-gamma (IFN-γ), as well as the master transcription factor of Tregs (Forkhead Box P3 (Foxp3)) and three genes related to oxidative stress reactions (inducible nitric oxide synthase (iNos), Superoxide dismutase (SOD) 1 and 2) were selected for qRT-PCR. Moreover, three potential nuclear encoded reference genes (beta-actin (ACTB), peptidylprolyl isomerase A (PPIA) and glyceraldehyde-3-phosphate dehydrogenase (GAPDH)) were included, which had been used in previous studies for this purpose (Borowska et al., 2016; Dreyling and Hasselmann, 2022). Primers were selected from previous studies or were designed based on reference sequences from the National Center for Biotechnology Information (NCBI) (Abdul-Careem et al., 2006, 2007; Dalgaard et al., 2015; Borowska et al., 2019; Cheng et al., 2020; French et al., 2020; Burkhardt et al., 2022; Dreyling and Hasselmann, 2022). A list of the final genes and related primers used in this work is provided in Table 1.

**Table 1:** Primer sequences for quantitative reverse transcription-PCR.

Gene		Primer Sequence	Product size (bp)	NCBI Accession Number
GAPDH	F	GAAGGCTGGGGCTCATCTG	150	NM_204305.1
	R	CAGTTGGTGGTGCACGATG		
ACTB	F	GCTGTGCTGTCCCTGTATGC	210	NM_205518
	R	TTCTCTCTCGGCTGTGGTGG		
PPIA	F	TGACTTTACGCGCCACAACG	165	NM_001166326
	R	TCGGTCTTGGCAGTGCAGAT		
IL-1β	F	TCCTGGAGGAGGTTTTTGAG	93	NM_204524.2
	R	AGGACTGTGAGCGGGTGTAG		
IL-2	F	CCGTGGCTAACTAATCTGCTG	123	AF000631.1
	R	AACGTACATTTTGAGCCCGTA		
IL-4	F	GGAGAGCATCCGGATAGTGAA	194	AJ621249.1
	R	TCAGGAGCTGACGCATGTTG		
IL-6	F	TCTGTTCGCCTTTCAGACCTA	142	HM367074.1
	R	GACCACCTCATCGGGATTTAT		
IL-10	F	CGGGAGCTGAGGGTGAAGT	119	NM_001004414.4
	R	CAGCCAAAGGTCCCCTTAAAC		
IL-12	F	TGAAGGAGTTCCCAGATGC	152	AY262752
	R	CGTCTTGCTTGGCTCTTTATAG		
IL-13	F	ATGACACCAGAGTGGCACAAG	187	AJ621250.1
	R	TAGATCTCATTGTCGGTGCTCG		
IL-17	F	GAAGGTGATACGGCCAGGAC	260	NM_204460.2
	R	AGTTCACGCACCTGGAATGG		
TNF-α	F	CGCTCAGAACGACGTCAA	116	MF000729
	R	GTCGTCCACACCAACGAG		
IFN-γ	F	CTGAAGAACTGGACAGAGAG	264	NM_205149.1
	R	CACCAGCTTCTGTAAGATGC		
SOD1	F	TGGAGACAACACAAATGGGTGTA	94	NM_205064.1
	R	CACGTGCCTATCTGCATCTTTT		
SOD2	F	CCCACATCAGTGCAGAGATCA	124	NM_204211
	R	TGAGCTGTAACATCACCTTTTGC		
iNos	F	TGACTTGGAAAAGAAAGGGATCAA	169	NM_204961.2
	R	GACTTTAGGCTGCCCAGGTTG		
Foxp3	F	AGTACGCCACAACCTGAGCCT	134	MT133687
	R	ACTTCAGGTCGCAGTCCCTG		

Confirmation of primer specificity was performed in a standard PCR, using DreamTaq Green (#K1081; Thermo Fisher Scientific, Waltham, MA) according to manufacturer's instructions, with the same conditions that were used for the final analysis. Standard agarose gel-electrophoresis was used to visualize resulting PCR products, which were then further purified and sequenced bidirectionally by Sanger sequencing technique (performed by Microsynth AG, Balgach, Switzerland).

Assay performance was evaluated on a Biomark HD using Flex Six™ Gene Expression Integrated Fluidic Circuits (IFC) (both Standard Biotools, South San Francisco, CA). Previously sequenced PCR products were used for a 10-step 10-fold dilution series, starting with a concentration of 25 ng/µL, to determine limits of detection, linear dynamic detection range, variation at detection limit, PCR efficiency, and specific melting curves of each product, respectively (via standard curve as described in Bustin et al. (2009)). Efficiency was above 90% for 16 primers and above 80% for one primer. Melting and standard curves are provided in the supplementary file S2.

Gene expression analysis was performed on Biomark HD (Standard Biotools, South San Francisco, CA) according to the manufacturer’s protocol. In order to remove remaining DNA from RNA samples, 2 µg RNA extract was digested using DNase I (#EN0525, Thermo Fisher Scientific, Waltham, MA). Reverse transcription was performed with Reverse Transcription Master Mix (#100-6297, Standard Biotools, South San Francisco, CA), containing a mixture of poly-T and random oligonucleotides, with the input of 1 µL DNase-treated RNA. Generated cDNA samples were stored at – 20°C until further processing. With a pool of all primers used for the final qPCR runs, a pre-amplification step was performed using the Preamp Master Mix (#100-5744, Standard Biotools, South San Francisco, CA) and 1.25 µL cDNA with 15 cycles (for spleen) or 13 cycles (for CT). Primers were removed by exonuclease I digestion (New England Labs, Ipswich, MA) and samples were diluted fivefold.

The final qPCR runs were performed on a 96.96 Dynamic Array™ IFC for Gene Expression (Standard Biotools, South San Francisco, CA), using the Delta Gene Assays protocol with the manufacturer’s standard protocol for fast PCR and melting curve (see Table 1 supplementary file S1). IFC sample inlets were loaded with 5 µL of an exo I-treated, pre-amplified and diluted sample mixed with SsoFast™ EvaGreen® Supermix with Low ROX (#1725211, Bio-Rad Laboratories GmbH, Feldkirchen, Germany) and DNA Binding Dye Sample Loading Reagent (#100-3738, Standard Bio Tools, South San Francisco, CA). Primers were diluted with Assay Loading Reagent (#85000736, Standard Bio Tools, South San Francisco, CA) and DNA Suspension Buffer (#12090015, Thermo Fisher Scientific, Waltham, MA) to a final concentration of 5 μM and 5 µL of each primer dilution were loaded into the assay inlets in triplicate. In order to test for contamination of the primers and reagents, a negative control was included at all preparation steps.

Data evaluation and quality control was performed using the Standard Biotools Real-Time PCR analysis software (version 1.0.2). In addition to the automatic quality check of the software, the data were evaluated by eye. The peak ratio threshold was set to 0.8 and the quality threshold to 0.65. Only quantification cycles (Cq) from reactions with logarithmic increase of fluorescence and specific melting temperature were used. Normfinder was used to evaluate stability of the three potential reference genes for normalization (ACTB, PPIA, and GAPDH) (Andersen et al., 2004). Normalization was tested for strain, period, and diet in each tissue. For spleen, a combination of GAPDH and PPIA, and for the CT, a combination of GAPDH and ACTB was identified as the best reference genes for normalization under the experimental conditions.

Means of triplicates were calculated for all samples. For samples that only had 2 or 1 successful triplicate, that specific run was used. The Cq values of genes of interest were normalized to the average Cq values of the reference genes resulting in ΔCq values. ΔCq values were calculated as follows: ΔCq = mean Cq _(gen of interest)_ – mean Cq _(reference genes)_.

### Statistical analyses

Statistical analyses were performed with SAS Version 9.4 (SAS Institute Inc., Cary, NC) using a linear mixed model with the PROC MIXED method. Logarithmic, square root or squared transformations were used to stabilize variance and to meet the distributional assumptions. The individual hen was considered as the experimental unit. The following model was used:

Y_ijklm_ = µ + α_i_ + β_j_ + γ_k_ + (αβ)_ij_ + (αγ)_ik_ + (βγ)_jk_ + (αβγ)_ijk_ + δ_1_ + φ_m_ + ε_ijklm_,

where Y_ijklm_ = response variable, µ = overall mean, α_i_ = effect of strain (fixed), β_j_ = effect of hen production period (fixed), γ_k_ = effect of mineral P supplementation, all 2- and 3-fold-interactions among strain, production period and mineral P supplementation (fixed), δ_1_ = block (random), φ_m_ = father/rooster (random); and ε_ijklmn_ = residual error.

The sampling time of blood from the vena ulnaris was included as a covariate in order to consider probable effects of sampling duration on immune parameters. For splenocyte proliferation and antibody ELISA data, the respective well plate during analysis was included in the model as a further random effect. Covariables were checked for significance, and non-significant covariables were excluded from the model. Statistical significance was declared at P < 0.05. Results are generally presented as Least Squares Means (LSmean) and Standard Error of the Mean (SEM) of untransformed or retransformed data. However, for the qPCR results, LSmeans of ΔCq values were transformed using the 2^-ΔCq^ to facilitate interpretation and graphical presentation. The corresponding LSmeans and SEM reflect this transformation. Residuals were tested for normal distribution and homogenous error variance via graphical check of residual plots (Kozak and Piepho, 2018).

Genes which were only expressed in a minor proportion of the samples during the gene expression analysis were analysed for their expression frequency, using a generalised linear model with the PROC GLIMMIX method and logit link function. Binomial distribution was assumed. Originally continuous expression data were converted into binary variables (expressed: yes/no) in order to adequately model the small number of cases. The underlying model structure was based on the linear mixed model of the other evaluations. In order to enable a stable parameter estimation in the very small numbers of cases, the model was simplified by removing 3-fold interactions and considering only main effects and 2-fold interactions. Model quality was assessed using the ratio of Pearson Chi² to the degrees of freedom (Chi²/df). If the Pearson Chi² to degrees of freedom ratio indicated over- or underdispersion, additional dispersion parameters were estimated. Statistical significance was declared at P < 0.05. Results are presented as LSmeans and SEM.

## Results

To note, as this research article focuses on the effects of a total renunciation of dietary mineral P on immune traits, in the following the results regarding the main effect of P as well as the interaction effects of P with either strain or period or both will be shown and discussed. The main effects of period and the interaction effects of period and strain are addressed in a separate publication, as these relate to a different thematic focus.

### Impact of dietary phosphorus supplementation and genetic background on the distribution and total cell count of immune cells in blood and lymphatic tissue

In order to investigate an influence of a complete renunciation of dietary mineral P on the systemic and gut-associated immune system, the numbers and frequency of various immune cell types and subtypes were investigated in blood, spleen and CT.

*Blood.* In the blood, the interactions of the treatments (P × strain × period, P × period or P × strain) had no effect, neither on the total cell counts per µL blood (Table 2), nor on the relative proportion of immune cell subsets within total leukocytes (see Table 2 supplementary file S3). When fed the P- diet compared to the P+ diet, lower total numbers of leukocytes (F(1,43.1) = 7.83, P = 0.008), total T cells (F(1,45.1) = 5.81, P = 0.02) and CD4^+^ T cells (F(1,46.7) = 4.41, P = 0.041) were found in hens of both strains, whereas no effect of the diet was reflected within cell frequencies. Regarding the strains, LB hens had higher numbers of thrombocytes (F(1,16.7) = 19.86, P < 0.001), monocytes (F(1,17.3) = 4.73, P = 0,044), heterophils (F(1,69) = 103.21, P < 0.001) and CD4^+^ T cells (F(1,17.2) = 6.55, P = 0.02), as well as a higher heterophil to lymphocyte ratio (F(1,69) = 86.56, P < 0.001) than LSL hens. LSL hens had higher numbers of γδ T cells (F(1,17.6) = 6.70, P = 0.019) and B cells (F(1,18.3) = 6.33, P = 0.021) than LB hens. Moreover, effects of strain were evident in the proportion of immune cells within total leukocytes. LB hens had a higher relative proportion of heterophils (F(1,71) = 119.95, P < 0.001), whereas LSL hens had higher proportions of total T cells (F(1,18.2) = 5.32, P = 0.033), cytotoxic T cells (F(1,71) = 5.57, P = 0.021), γδ T cells (F(1,17.3) = 20.31, P < 0.001) and B cells (F(1,17.9) = 11.81, P = 0.003).

**Table 2:** Impact of dietary P, strain and period on the number of immune cells per µL blood (× 10^3^) and heterophil-to-lymphocyte (H:L) ratio of laying hens.

Dietary P^1^	Strain^2^	Leukocytes	Thrombocytes	Monocytes	Heterophils	Total T Cells	Cytotoxic T Cells	CD4^+^ T Cells	γδ T Cells	B Cells	H:L Ratio
P-	LB	32.4 ± 1.2	49.4 ± 1.8	1.68 ± 0.1	5.15 ± 0.4	15.3 ± 0.8	3.34 ± 0.2	7.93 ± 0.4	4.74 ± 0.3	2.0 ± 0.2	0.3 ± 0.03
P-	LSL	30.4 ± 1.2	41.1 ± 1.8	1.37 ± 0.1	2.31 ± 0.2	14.8 ± 0.8	3.41 ± 0.2	6.18 ± 0.4	5.40 ± 0.3	2.74 ± 0.3	0.1 ± 0.01
P+	LB	34.8 ± 1.2	50.6 ± 1.8	1.84 ± 0.2	6.13 ± 0.5	15.9 ± 0.8	3.41 ± 0.2	8.08 ± 0.4	4.87 ± 0.3	2.48 ± 0.2	0.3 ± 0.03
P+	LSL	32.9 ± 1.2	42.7 ± 1.8	1.55 ± 0.1	2.62 ± 0.2	17.1 ± 0.8	3.82 ± 0.2	7.35 ± 0.4	6.32 ± 0.3	3.07 ± 0.3	0.1 ± 0.01
p-values^3^											
P × Strain × Period		0.486	0.605	0.311	0.566	0.123	0.659	0.232	0.100	0.189	0.216
P × Period		0.982	0.688	0.439	0.136	0.205	0.602	0.679	0.278	0.136	0.067
P × Strain		0.956	0.897	0.828	0.775	0.157	0.306	0.113	0.166	0.612	0.516
P		0.008	0.394	0.125	0.07	0.020	0.138	0.041	0.069	0.089	0.614
		P- < P+				P- < P+		P- < P+			
Strain		0.179	< 0.001	0.044	< 0.001	0.757	0.345	0.020	0.019	0.021	< 0.001
			LB > LSL	LB > LSL	LB > LSL			LB > LSL	LB < LSL	LB < LSL	LB > LSL

Data are presented as LSmeans ± SEM.

^1^P- = without mineral P supplementation, P+ = supplemented with 1 g P / kg feed.

^2^LB = Lohmann Brown-Classic, LSL = Lohmann LSL-Classic.

^3^Results of statistical analysis of interactions (P × strain × period, P × period, P × strain) and main effects of P or strain using a linear mixed model are given. In case of significance (P < 0.05), significant differences of the post hoc test (Fisher LSD; P < 0.05) and direction of effects are stated below.

*Spleen.* Neither total cell counts per g spleen (Table 3 supplementary file S3), nor the relative proportion (Table 4 supplementary file S3) of the investigated immune cell subsets were affected by any interactions of the treatments (P × strain × period, P × period or P × strain), nor by the dietary mineral P concentration. Cell counts per g tissue of almost all subpopulations were affected by strain, with higher numbers in LSL hens compared to LB hens. This included leukocytes (F(1,59.6) = 46.58, P < 0.001), macrophages (F(1,60.3) = 46.35, P < 0.001), NK cells (F(1,69) = 23.27, P < 0.001), total T cells (F(1,59.7) = 68.88, P < 0.001), cytotoxic T cells (F(1,16.5) = 32.23, P < 0.001), total CD4^+^ T cells (F(1,17) = 8.50, P = 0.01), γδ T cells (F(1,17.8) = 73.82, P < 0.001), CD4^+^CD25^-^ T cells (F(1,16.8) = 7.47, P = 0.014) and CD4^+^CD25^dim^ T cells (F(1,59.6) = 6.06, P = 0.017). In addition, LSL hens (1.76 g ± 0.06) had lower spleen weights compared to LB hens (2.24 g ± 0.08) (F(1,18) = 4.66, P < 0.001), and showed a lower total body weight compared to LB hens (LSL: 1.299 kg ± 0.016, LB: 1.613 kg ± 0.016) (F(1,72) = 189.91; P < 0.001). No difference was found in the relative spleen weight (F(1,18.2) = 0.22, P = 0.643). Moreover, LB hens had higher proportions of total CD4^+^ T cells (F(1,17.4) = 5.25, P = 0.035), B cells (F(1,69) = 33.23, P < 0.001) and CD4^+^CD25^high^ T cells (F(1,15) = 11.24, P = 0.004). LSL hens had higher proportions of macrophages (F(1,16.1) = 17.34, P < 0.001), NK cells (F(1,60.4) = 5.97, P = 0.018), total T cells (F(1,18.6) = 16.57, P < 0.001) and γδ T cells in total (F(1,18.4) = 16.51, P < 0.001).

*Cecal Tonsils.* Within the CT, cell counts per g CT of CD4^+^CD25^dim^ T cells (F(1,41.2) = 4.89, P = 0.033) and the relative proportion of NK (F(1,52) = 5.87, P = 0.019) were affected by 3-fold interaction (P × strain × period) (Table 5 supplementary file S3). When fed the P- diet, numbers of CD4^+^CD25^dim^ T cells were higher in LSL hens compared to LB hens only at an age of 24 wk (t(46.7) = -3.13, P = 0.003) and not at an age of 19 wk (t(44.7) = -1.27, P = 0.211), whereas at an age of 19 wk the same effect was apparent in the P+ diet (LSL > LB; t(46.6) = -2.37, P = 0.022). An effect of mineral P supply on the frequency of NK cells among leukocytes was found to be also related to period and strain, with higher frequencies in the P- diet vs. the P+ diet in 24 wk old LB hens (t(52.6) = 2.27, P = 0.027) and 19 wk old LSL hens (t(51.4) = 2.98, P < 0.004). Moreover, higher frequencies were also observed in LSL hens at 19 wk vs. 24 wk of age (t(51.4) = 3.56, P < 0.001) when fed the P- diet only. Relative proportions of NK cells were also higher in LB hens compared to LSL hens at both periods when fed the P+ diet (19 wk: t(68.9) = 4.35, P < 0.001; 24 wk: t(68.6) = 4.93, P < 0.001), but only in wk 24 when fed the P- diet (19 wk: t(68.6) = 1.63, P = 0.108; 24 wk: t(68.9) = 6.62, P < 0.001).

No further impact of interactions of the treatments (P × period or P × strain), and no main effects of the dietary P treatments on the total cell counts per g CT were found (Table 3). However, the relative proportion of immune cell subsets among leukocytes was affected by dietary P (Table 6 supplementary file S3). Hens fed the P- diet had a higher proportion of NK cells (F(1,52.1) = 7.97, P = 0.007) and CD4^+^CD25^high^ T cells (F(1,52.8) = 5.10, P = 0.028) compared to hens fed the P+ diet. In addition, strain affected the frequency among leukocytes as well as number per g CT in a variety of leukocytes subsets. LSL hens had higher numbers of γδ T cells (F(1,13.4) = 32.35, P < 0.001), B cells (F(1,16) = 7.99, P = 0.012), CD4^+^CD25^dim^ T cells (F(1,13.7) = 7.13, P = 0.019) and CD4^+^CD25^high^ T cells (F(1,12.3)= 11.81, P = 0.005). LB hens had higher numbers of NK cells (F(1,61.3)= 20.64, P < 0.001). Moreover, LB hens (0.28 g ± 0.01) had higher CT weights compared to LSL hens (0.2 g ± 0.01) (F(1,60.9) = 6.88, P < 0.001). LSL hens had a higher frequency of γδ T cells (F(1,70) = 31.61, P < 0.001), B cells (F(1,17.9) = 8.98, P = 0.008) and CD4^+^CD25^high^ T cells (F(1,17.9) = 11.72, P = 0.003), whereas LB hens had higher proportions of NK cells (F(1,17.3) = 62.62, P < 0.001) and CTLs (F(1,17.4) = 41.33, P < 0.001.

**Table 3:** Impact of dietary P, strain and period on the number of immune cells (× 10^6^) per g cecal tonsil of laying hens.

Dietary P^1^	Strain^2^	Leukocytes	NK Cells	Total T Cells	Cytotoxic T Cells	CD4^+^ T Cells	γδ T Cells	B Cells	CD4^+^CD25^-^ T Cells	CD4^+^CD25^dim^ T Cells	CD4^+^CD25^high^ T Cells
P-	LB	31.0 ± 3.7	0.76 ± 0.1	23.6 ± 2.8	10.9 ± 1.4	6.37 ± 0.8	4.80 ± 0.6	5.43 ± 0.8	3.97 ± 0.6	1.53 ± 0.2	0.73 ± 0.1
P-	LSL	39.0 ± 4.6	0.53 ± 0.1	29.0 ± 3.4	10.6 ± 1.4	7.83 ± 1.0	8.66 ± 1.1	8.82 ± 1.3	4.32 ± 0.6	2.29 ± 0.3	1.05 ± 0.1
P+	LB	33.6 ± 4.0	0.69 ± 0.1	25.8 ± 3.1	12.9 ± 1.7	5.84 ± 0.8	5.24 ± 0.7	5.79 ± 0.9	3.63 ± 0.5	1.52 ± 0.2	0.59 ± 0.1
P+	LSL	37.2 ± 4.4	0.40 ± 0.1	27.2 ± 3.2	10.1 ± 1.3	7.64 ± 1.0	7.73 ± 0.9	8.74 ± 1.3	4.49 ± 0.6	2.02 ± 0.3	0.98 ± 0.1
p-values^3^											
P × Strain × Period		0.438	0.319	0.590	0.901	0.212	0.826	0.149	0.371	0.033^4^	0.840
P × Period		0.128	0.178	0.157	0.391	0.188	0.138	0.171	0.288	0.136	0.374
P × Strain		0.420	0.373	0.297	0.182	0.730	0.231	0.769	0.550	0.426	0.456
P		0.836	0.072	0.851	0.485	0.535	0.877	0.821	0.807	0.367	0.158
Strain		0.145	< 0.001	0.241	0.324	0.069	< 0.001	0.012	0.268	0.019	0.005
			LB > LSL				LB < LSL	LB < LSL		LB < LSL	LB < LSL

Data are presented as LSmeans ± SEM.

^1^P- = without mineral P supplementation, P+ = supplemented with 1 g P / kg feed.

^2^LB = Lohmann Brown-Classic, LSL = Lohmann LSL-Classic.

^3^Results of statistical analysis of interactions (P × strain × period, P × period, P × strain) and main effects of P or strain using a linear mixed model are given. In case of significance (P < 0.05), significant differences of the post hoc test (Fisher LSD; P < 0.05) and direction of effects are stated below.

^4^Significant interactions (P × strain × period) are summarized in Table 4 supplementary file S3.

### Impact of dietary phosphorus supplementation and genetic background on immune cell functionality

*Proliferation capacity of splenic lymphocytes.* Splenocyte proliferation capacity was neither affected by interactions of the treatments (P × strain × period, P × period or P × strain), nor by any single main effects of dietary P or strain.

*Antibody concentration.* No interactions of the treatments (P × strain × period, P × period or P × strain), were observed for antibody concentrations in plasma and bile. There was no impact of dietary P or strain on plasma IgY, plasma IgM or IgA in bile. Plasma IgA was affected by strain, with higher antibody concentrations in LB hens compared to LSL hens (F(1,17.8) = 40.22, P < 0.001) (Table 7 supplementary file S3).

### Impact of dietary phosphorus supplementation and genetic background on gene expression related to immunological pathways

As a measure of inflammatory conditions, we assessed the gene expression of pro- and anti-inflammatory cytokines as well as the expression of genes related to oxidative stress reactions in spleen and CT under the presumed impact of a complete renunciation of dietary mineral P.

*Spleen.* Gene expression was not impaired by interactions of the treatments (P × strain × period, P × period or P × strain), nor by the main effect of dietary P. However, the strain affected the gene expression, with higher expression levels of IL-1β (F(1,70) = 79.17, P < 0.001), TNF-α (F(1,71) = 42.71, P < 0.001) and iNos (F(1,17.2) = 20.54, P < 0.001) in LSL hens and higher expression of IFN-γ (F(1,15.2) = 13.01, P = 0.003) in LB hens (Table 8 supplementary file S3).

*Cecal Tonsils.* Gene expression was not affected by the interaction of P × strain × period. However, interaction of P × strain affected the expression of IL-1β (F(1,43.2) = 4.69, P = 0.036), with higher expression found in hens fed the P- diet compared to the P+ diet only in the LSL strain (t(43.2) = -2.21, P = 0.032), but not in the LB strain (t(43.2) = 0.85, P = 0.401). In addition, higher expression levels were found in LSL hens fed the P- diet compared to LB hens with the same diet (t(43.3) = 3.77, P < 0.001), but not when fed the P+ diet (t(43.3) = 0.96, P = 0.342). Likewise, expression frequency of IL-4 (F(1,46.83) = 8.04, P = 0.007) was affected. LSL hens fed the P- diet showed a lower IL-4 frequency compared to LB hens fed the P- diet (t(55.8) = 2.67, P = 0.009) or LSL hens fed the P+ diet (t(47.86) = -2.55, P = 0.014) (Table 4).

**Table 4:** Impact of dietary P, strain and period on gene expression of pro- and anti-inflammatory cytokines, foxp3 and genes related to oxidative stress reactions in the cecal tonsil of laying hens.

Dietary P^3^	Strain^4^	IL-1β	IL-2	IL-6	IL-10	IL-12	IL-13	IL-17
		[2^-ΔCq^ × 10^-4^]^1^						
P-	LB	8.0 ± 0.7^a^	11.1 ± 0.6	0.17 ± 0.03	0.63 ± 0.1	0.45 ± 0.05	0.74 ± 0.1	4.6 ± 0.7
P-	LSL	12.5 ± 1.1^b^	11.7 ± 0.7	0.16 ± 0.03	0.99 ± 0.1	0.54 ± 0.05	0.66 ± 0.1	4.3 ± 0.7
P+	LB	8.8 ± 0.8^ab^	10.8 ± 0.6	0.17 ± 0.03	0.66 ± 0.1	0.48 ± 0.05	0.56 ± 0.1	5.3 ± 0.8
P+	LSL	9.8 ± 0.9^a^	11.2 ± 0.6	0.14 ± 0.02	0.86 ± 0.1	0.44 ± 0.04	0.78 ± 0.1	3.5 ± 0.5
p-values^5^								
P × Strain × Period		0.288	0.662	0.604	0.474	0.168	0.464	0.191
P × Period		0.053	0.007^6^	0.025^6^	0.016^6^	0.020^6^	0.644	0.423
P x Strain		0.036	0.898	0.641	0.391	0.146	0.137	0.178
P		0.340	0.469	0.626	0.662	0.478	0.706	0.775
Strain		0.006	0.392	0.471	0.006	0.712	0.451	0.144
		LB < LSL			LB < LSL			


Dietary P^3^	Strain^4^	TNF-α	IFN-γ	SOD1	SOD2	iNos	Foxp3	IL-4^2^
		[2^-ΔCq^ × 10^-4^]^1^						[%/100]
P-	LB	6.35 ± 0.5	3.02 ± 0.3	962 ± 38	309 ± 11	80.8 ± 3.7	43.9 ± 3.9	0.44 ± 0.15^a^
P-	LSL	8.19 ± 0.6	2.08 ± 0.2	901 ± 37	267 ± 9	105.5 ± 4.7	58.1 ± 5.1	0.04 ± 0.03^b^
P+	LB	6.93 ± 0.6	3.45 ± 0.3	861 ± 36	284 ± 10	86.4 ± 4.0	51.7 ± 4.5	0.26 ± 0.12^ab^
P+	LSL	7.72 ± 0.6	1.70 ± 0.2	897 ± 37	267 ± 9	94.8 ± 4.4	53.5 ± 4.7	0.34 ± 0.14^a^
p-values^5^								
P × Strain × Period		0.921	0.477	0.948	0.475	0.492	0.760	n.d.
P × Period		0.205	0.111	0.685	0.325	0.242	0.126	0.798
P x Strain		0.360	0.065	0.153	0.176	0.061	0.165	0.007
P		0.874	0.697	0.116	0.156	0.665	0.650	0.129
Strain		0.025	< 0.001	0.726	0.003	0.001	0.077	0.101
		LB < LSL	LB > LSL		LB > LSL	LB < LSL		

^1^Data are presented as 2^-ΔCq^-transformed LSmeans ± SEM of ΔCq values, with ΔCq = mean Cq (gen of interest) – mean Cq (reference genes).

^2^For IL-4, relative proportion [%] of expression frequency is given (n=80). In order to stabilize parameter estimation, the model was simplified by removing 3-fold interactions.

^3^P- = without mineral P supplementation, P+ = supplemented with 1 g P / kg feed.

^4^LB = Lohmann Brown-Classic, LSL = Lohmann LSL-Classic.

^5^Results of statistical analysis of interactions (P × strain × period, P × period, P × strain) and main effects of P or strain using a linear mixed model or generalised linear model are given. Directions of main effects are indicated when P < 0.05. In the case of interactions (P x strain) different superscript letters indicate differences in means (Fisher LSD; P < 0.05).

^6^Significant interactions (P × period) are summarized in Table 8 supplementary file S3.

The 2-fold interaction of P × period affected expression levels of IL-2 (F(1,43) = 7.91, P = 0.007), IL-6 (F(1,48.5) = 5.35, P = 0.025), IL-10 (F(1,51.9) = 6.15, P = 0.016) and IL-12 (F(1,50.5) = 5.75, P = 0.02) (Table 9 supplementary file S3). Only when fed the P+ diet, 19 wk old hens showed a higher expression of IL-2 (t(43) = -2.63, P = 0.012), IL-6 (t(48.5) = -2.24, P = 0.03) and IL-10 (t(51.9) = -2.43, P = 0.019) vs. 24 wk old hens. Moreover, expressions of IL-2 (t(43) = -2.51, P = 0.016) and IL-10 (t(51.9) = -2.06, P = 0.044) were higher with the P- diet vs. the P+ diet, but only at the age of 24 wk. Contrary, expression of IL-12 was higher when fed the P- diet vs. the P+ diet, but only in 19 wk old hens (t(50.5) = -2.20, P = 0.032). No main effects of the dietary P treatments on any of the analysed gene expressions in the CT were found. The strain affected expressions of IL-1β (F(1,17.1) = 9.63, P = 0.006), IL-10 (F(1,13.6) = 10.77, P = 0.006), TNF-α (F(1,72) = 5.27, P = 0.025), IFN-γ (F(1,18) = 24.76, P < 0.001), SOD2 (F(1,16.6) = 11.61, P = 0.003) and iNos (F(1,18) = 14.96, P = 0.001). Higher expressions of IL-1 β, IL-10, TNF-α and iNos were found in LSL hens, whereas LB hens had higher levels of IFN-γ and SOD2 (Table 4).

## Discussion

The results of the present study showed that the peripheral and gut-associated immune system of laying hens are sensitive to a renunciation of mineral P in the feed, especially T cell subsets. The renunciation of mineral P led to decreased numbers of T cells in the blood and an increased proportion of CD4^+^CD25^high^ T cells and NK cells in the CT. The CD4^+^CD25^high^ T cells are considered to enclose regulatory T cells in avian species (Burkhardt et al., 2022), which are one key cell type promoting anti-inflammatory immune regulation. Regulatory T cells express anti-inflammatory cytokines such as IL-10 in order to diminish proinflammatory immune mechanisms. Moreover, they regulate and prevent overreactions of the immune system by suppressing the activity of other immune cells. They are therefore of particular importance for immune tolerance, especially in the intestine (Schmidt et al., 2012; Burkhardt et al., 2022). Likewise, NK cells can have immunomodulatory properties. Besides their cytotoxic effector functions, NK cells modulate various immune cells, such as dendritic cells, T cells, B cells, and endothelial cells (Vivier et al., 2008; Meijerink et al., 2021). In addition, a higher expression of IL-2 and IL-10 in the CT of hens fed the P- diet, at onset of egg laying in wk 24, when the demand for P should differ from the demand before sexual maturation, mirrors the changes observed in the immune cell composition. The higher expression of IL-10 is consistent with the increased proportion of regulatory T cells. Moreover, IL-10 can induce NK cell cytotoxic activity (Carson et al., 1995; Mocellin et al., 2004; Lu et al., 2023). IL-2 is also known to promote NK cell cytotoxicity, proliferation and cytokine production, as well as T cell proliferation and differentiation, including regulatory T cells (Carson et al., 1995; Staeheli et al., 2001; Mocellin et al., 2004; Liao et al., 2013; Lu et al., 2023). Furthermore, we observed an increased expression of IL-12, a cytokine which is also known as NK cell stimulatory factor, in hens fed the P- diet, in wk 19, before the onset of egg laying (Trinchieri et al., 1992; Cho et al., 1996). In contrast, IL-6 showed a consistent expression when fed the P- diet, but was decreased when fed the P+ diet in wk 24, at the onset of egg laying. Although IL-6 is often associated with pro-inflammatory activities, it also plays an important role in maintaining mucosal integrity and intestinal immune defence (Dann et al., 2008; Kuhn et al., 2014) and has regenerative and anti-inflammatory properties (Scheller et al., 2011).

Taken together, our findings suggest that the absence of mineral P in the feed improves immune regulation, particularly with regard to a probably altered immunocompetence and increased metabolic challenges, which are associated with the onset of egg laying (Wigley et al., 2005; Johnston et al., 2012; Gonzalez-Uarquin et al., 2021). Therefore, it can be speculated, that metabolic changes are better compensated within the P- group. The cytokine profile, with higher levels of IL-10, IL-2 and IL-6, in combination with higher proportions of CD4^+^CD25^high^ T cells and NK cells, suggests that the complete renunciation of mineral P leads to a better regulated and improved anti-inflammatory immune response in the CT. In this context, the reduced number of immune cells in the blood, especially T cell subsets, could be the result of a selective regulation of the activation and restraint of these cells in the CT, rather than from systemic suppression. Koutsos and Klasing, (2014) described that nutrient deficiencies in general must be severe to negatively compromise the immune system, whereas moderate food or nutrient restrictions are often thought to improve immunity. This view is consistent with our observations. The inorganic P levels in the blood plasma of hens from this study fed the P- diet were reduced (Sommerfeld et al. 2024). However, immune cell subpopulations in blood, spleen and CT, antibody concentrations and splenocyte proliferation capacity as well as the heterophil to lymphocyte ratio, which is known to be highly sensitive for stressors, were not negatively affected by the P- diet. Therefore, we suggest that the renunciation of mineral P from the feed did not represent a stressor for the immune system in our study.

The results of a companion study showed that the renunciation of mineral P led to a higher InsP_6_ degradation in the digestive tract compared to a diet with slightly reduced mineral P. Moreover, higher plasma MI concentrations were found in hens fed the P- diet, depending on age and strain (Sommerfeld et al., 2024). As already mentioned, degradation products such as lower InsP_x_ and MI take part in immune signalling cascades by serving as structural precursors for second messengers or acting as second messengers themselves (Croze and Soulage, 2013; Huber, 2016). Moreover, they are considered to have beneficial pathophysiological, immune regulatory and anti-inflammatory properties (Piranlioglu et al., 2019; Baldassarre et al., 2021). In a previous trial, a 20 % reduction in dietary mineral P supplementation was evaluated relative to a standard diet. We found no effects of such reduction on plasma inorganic P or MI concentrations and no major effects on performance or metabolic traits (Sommerfeld et al., 2020b), but increasing effects on some immune parameters (Hofmann et al., 2021). Hofmann et al. (2021) showed that the 20 % reduction of mineral P enhanced immune cell counts and antibody concentrations. The results of the present study, comparing the mineral-P-reduced diet of our former study (Hofmann et al., 2021) to a diet without any mineral P addition, indicate that the complete renunciation of mineral P resulted in lower blood cell counts and did not further improve antibody concentrations. This could be explained by a potentially different InsP_6_ degradation due to the different mineral P contents of the diets. Furthermore, nutrient composition can affect the intestinal microbiota, especially components that cannot be digested by the host itself (Klasing, 2007). Studies in pigs and poultry have demonstrated that adjustments in dietary P and calcium concentrations and InsP_6_ degradability shape the intestinal microbiota (Heyer et al., 2019; Roth et al., 2022; Rubio-Cervantes et al., 2025), and induce changes in the formation and composition of bacterial fermentation products (Tilocca et al., 2017). Therefore, it can be speculated that the renunciation of mineral P might have led to changes in the microbial composition and the abundance of certain bacterial fermentation products. Short chain fatty acids such as butyrate are known to be potent mediators of immune regulation (Bortoluzzi et al., 2017; Lee et al., 2018) and modulators of Tregs (Furusawa et al., 2013; Lee et al., 2018). Both mechanisms might fit into our findings that an increase in CD4^+^CD25^high^ T cells and cytokines promote a more anti-inflammatory response and improved immune regulation. The fact that these effects are limited to the CT and do not appear in the systemic immune system might be due to CT's proximity to the intestinal microbiota and the site of InsP_6_ degradation. However, it has to be considered that the feeding period of the experimental diets was limited to 4 wk. A prolonged exposure to a mineral P-free diet could either manifest the effects shown in this study or become a physiological challenge and an actual nutritional stressor. Further studies are needed to evaluate the effects of an extended absence of mineral P supplementation in the diet and to assess the impact of the renunciation of mineral P on the immune response to infection diseases.

The differences between the two laying hen strains were consistent with those found by Hofmann et al. (2021). However, despite their distinctive genetic differences in the immune system and metabolic pathways, hardly any parameters were influenced by the interaction of diet and strain. Nevertheless, immunological differences between the strains were highly pronounced and observed across almost all analysed immune parameters. LB hens generally showed higher immune cell numbers related to innate and unspecific immune responses, such as monocytes, heterophils and NK cells. They also had a higher plasma IgA concentration, and a higher heterophil to lymphocyte ratio. In accordance with this, LB hens showed a higher expression of IFN-γ, in spleen and CT, and a higher expression of SOD2 in the CT. IFN-γ is known to be a primary activator of macrophages and a potent inducer of nitric oxide, whereas SOD2 plays an important role in protecting against reactive oxygen species (Song et al., 1997; Bafana et al., 2011). The increased expression of SOD2 could be an adaptive mechanism to the macrophages and IFN-γ predominant immune response, as well as to an intensified oxidative stress reaction in LB hens. Moreover, when fed the P- diet, a higher number of LB hens showed an expression of IL-4 in the CTs, compared to LSL hens, which suggests that P- diets improved anti-inflammatory pathways especially in LB hens. In contrast, LSL hens had higher numbers and proportions of several T cell subpopulations such as cytotoxic T cells, CD4^+^ T cells, γδ T cells, CD4^+^CD25^dim^ T cells, CD4^+^CD25^high^ T cells and B cells. Moreover, LSL hens showed higher expression levels of pro-inflammatory IL-1β, TNF-α and iNOS, but also higher levels of the immune regulating cytokine IL-10. Additionally, in LSL hens IL-1β expression further increased, whereas IL-4 frequency decreased when fed the P- diet. Therefore, LSL hens may have a more pronounced specific and highly reactive, but also strictly regulated immune response, based on their cytokine profile. Similar differences between the 2 strains were described in previous studies, which also found strain-specific differences in the immune response, particularly a generally higher innate and humoral immune response in LB hens and a more cellular pronounced immunity in LSL hens (Habig et al., 2012; Hofmann et al., 2021).

## Conclusions

The results of the present study demonstrate that the systemic and gut-associated immune systems are sensitive to changes in the dietary mineral P composition. The complete renunciation of mineral P modified immune regulation within the gut-associated immune system and fostered an anti-inflammatory environment. However, further studies are required to evaluate the impact of the renunciation of mineral P on the immune response to infectious diseases, as well as its overall effect on chicken health. Moreover, it should be investigated how a prolonged absence of mineral P in the diet affects the immune system in order to determine whether positive effects are manifested or whether an extended absence negatively influences the immune system of hens, particularly during the egg laying period. Differences between the 2 laying hen strains were reconfirmed. The study also expanded our knowledge of differences regarding immune cell distribution by strain-specific cytokine profiles, with LB hens showing a stronger innate immune response based predominantly on macrophages and IFN-γ, while LSL hens expressed a more specific and reactive, but also strictly regulated immune response.

## CRediT authorship contribution statement

**Nadine Wallauch:** Writing – original draft, Visualization, Methodology, Investigation, Formal analysis. **Sonja Schmucker:** Writing – review & editing, Validation, Supervision, Methodology, Conceptualization. **Tanja Hofmann:** Writing – review & editing. **Vera Sommerfeld:** Writing – review & editing. **Martin Hasselmann:** Writing – review & editing, Methodology. **Korinna Huber:** Writing – review & editing, Conceptualization. **Markus Rodehutscord:** Writing – review & editing, Conceptualization. **Volker Stefanski:** Writing – review & editing, Supervision, Conceptualization.

## Disclosures

The authors declare that they have no known competing financial interests or personal relationships that could have appeared to influence the work reported in this paper.

## Appendix Supplementary materials

Image, application 1

Image, application 2

Image, application 3

## Footnotes

Supplementary material associated with this article can be found, in the online version, at doi:10.1016/j.psj.2026.106771.
