# Supplementary material for: Impact of feeding diets without mineral P supplement on the immune system of two laying hen strains
Source: Poult Sci. 2026 Mar 13;105(6):106771. doi: 10.1016/j.psj.2026.106771 (PMC13052099; doi:10.1016/j.psj.2026.106771)
Supplement: Supplementary file 1 [file mmc1.pdf]

S1 – Gating strategies of flow cytometric analysis and cycling conditions for qRT-PCR

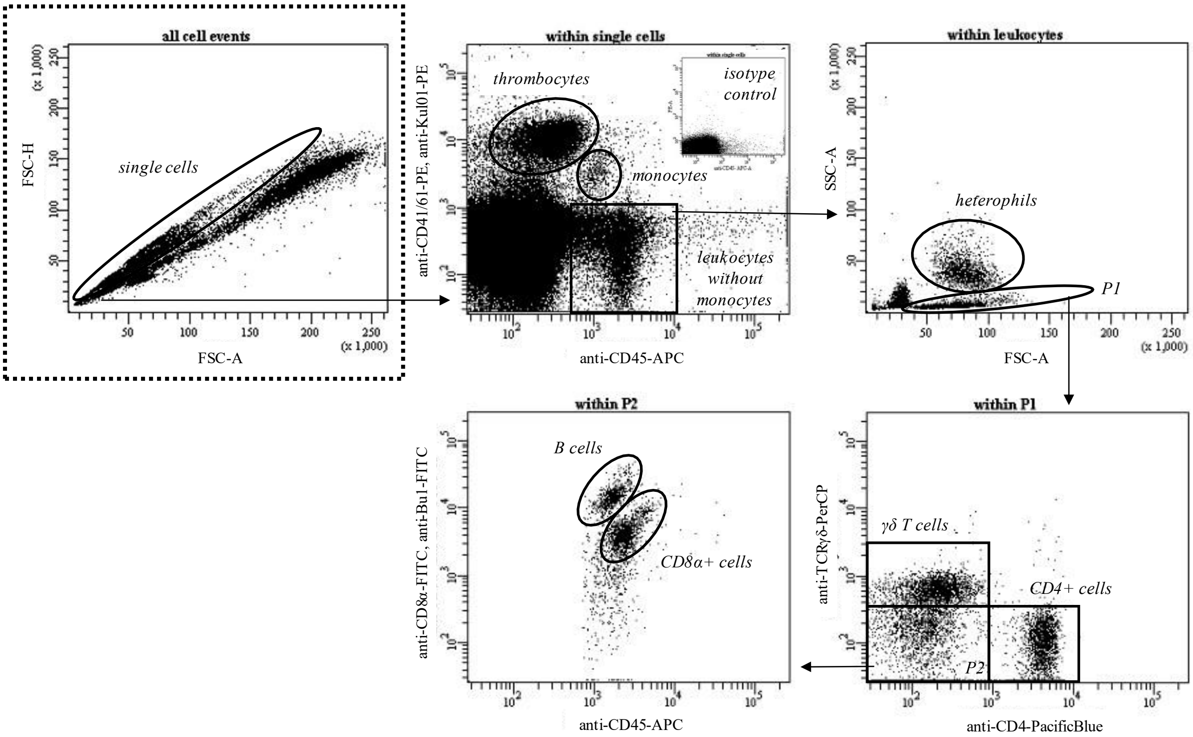

Figure 1 Gating strategy of the flow cytometric analysis of chicken whole blood. Representative dot plots from a Lohmann Brown-Classic hen; dashed line marks starting point of gating.

S1 – Gating strategies of flow cytometric analysis and cycling conditions for qRT-PCR

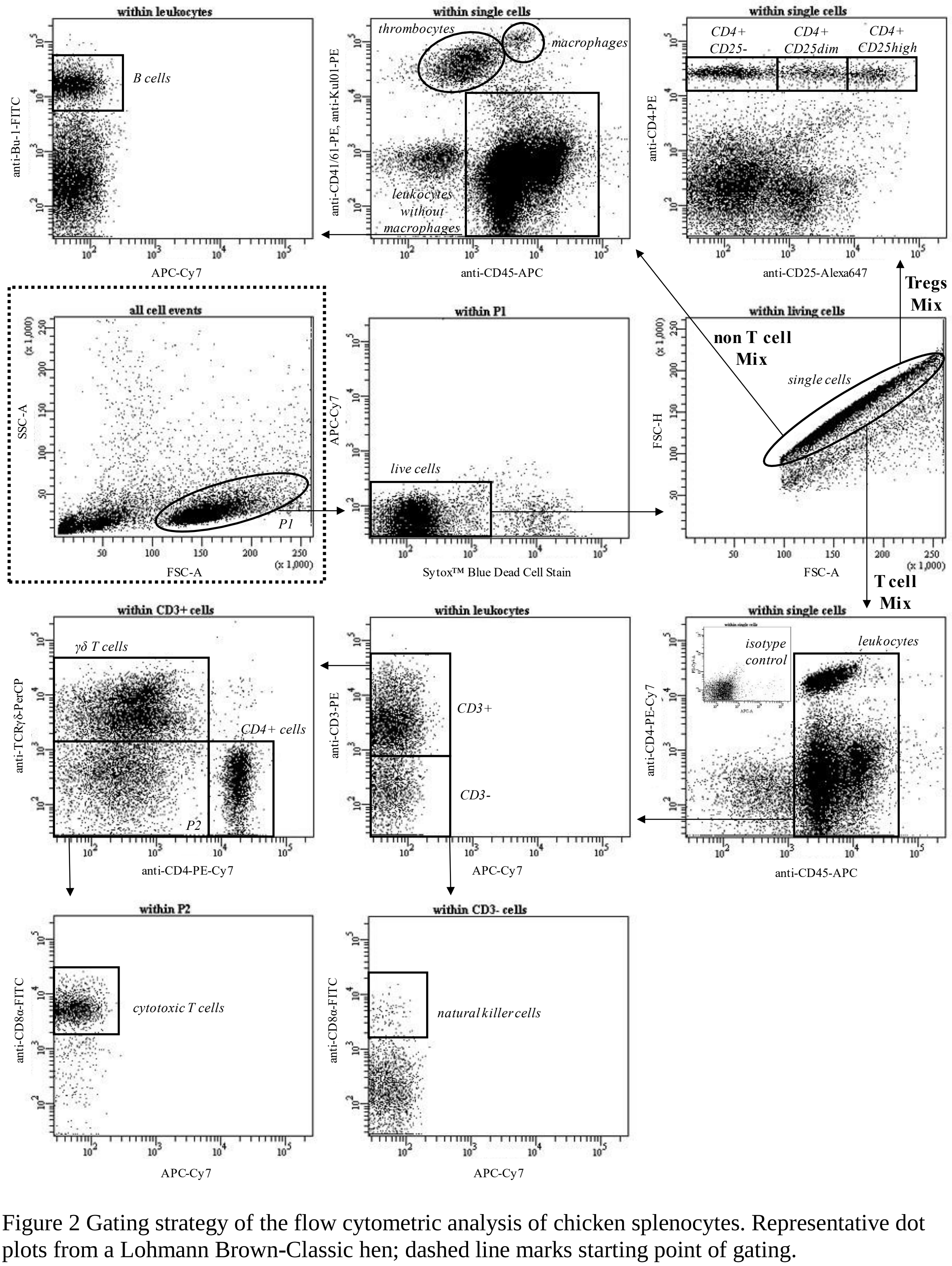

Figure 2 Gating strategy of the flow cytometric analysis of chicken splenocytes. Representative dot plots from a Lohmann Brown-Classic hen; dashed line marks starting point of gating.

S1 – Gating strategies of flow cytometric analysis and cycling conditions for qRT-PCR

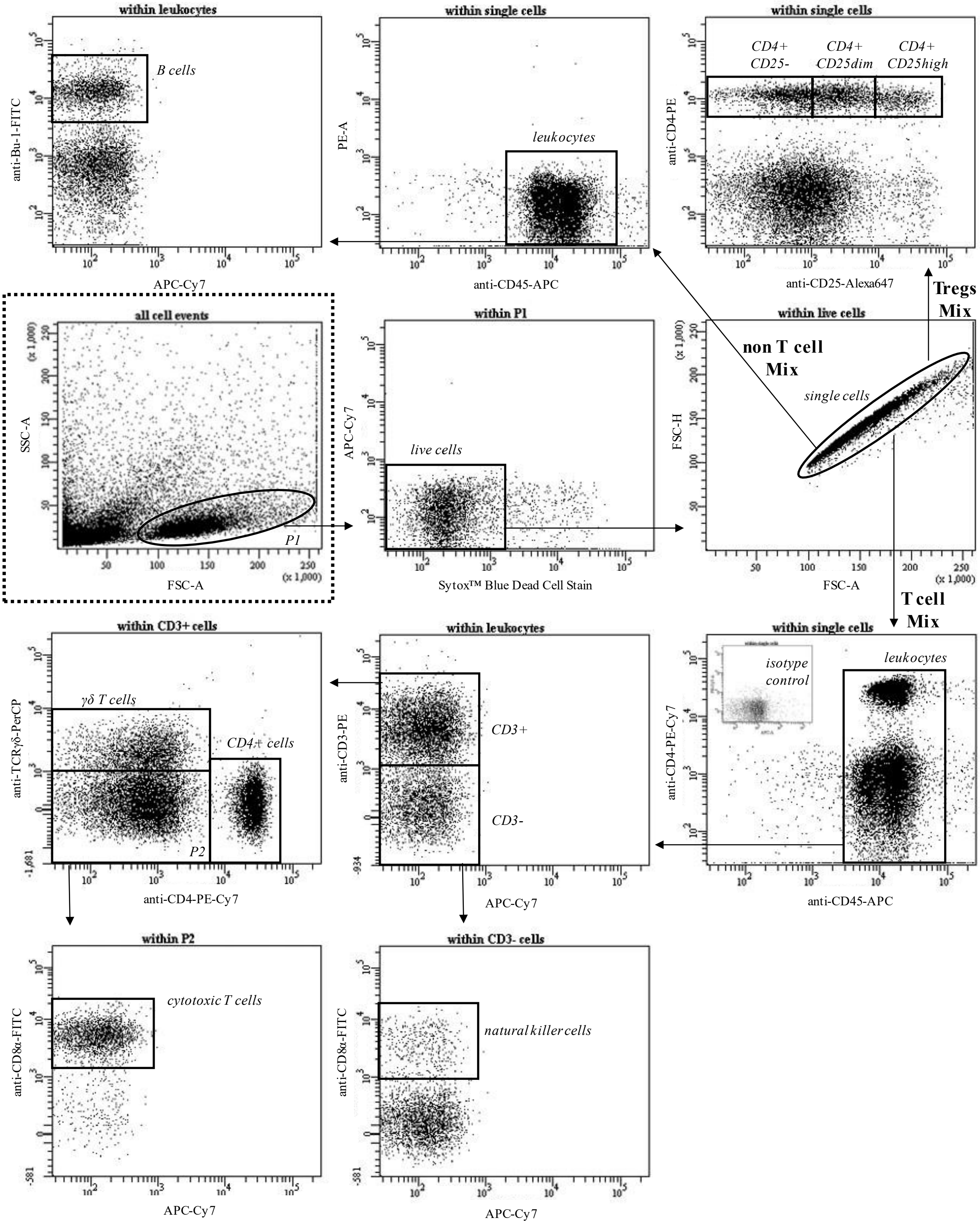

Figure 3 Gating strategy of the flow cytometric analysis of chicken IELs from cecal tonsils. Representative dot plots from a Lohmann Brown-Classic hen; dashed line marks starting point of gating.

S1 – Gating strategies of flow cytometric analysis and cycling conditions for qRT-PCR

Table 1 Thermal cycling conditions used on the final qPCR runs according to the manufacturers protocol (Standard Bio Tools)

Step	Flex Six		96.96 IFC	
	Temperature	Time	Temperature	Time
Thermal mixing	25 °C	30 min	70 °C	40 min
	70 °C	60 min	60 °C	30 sec
Hot start	95 °C	1 min	95 °C	1 min
PCR, 30 cycles	96 °C	5 sec	96 °C	5 sec
	60 °C	20 sec	60 °C	20 sec
Melting curve	60 °C	3 sec	60 °C	3 sec
	60-95 °C	1 °C/3 sec	60-95 °C	1 °C/3 sec

[1]Flex Six™ Gene Expression Integrated Fluidic Circuits (Standard Bio Tools).

[2]96.96 Integrated Fluidic Circuits (**IFC**), 96.96 Dynamic Array™ IFC for Gene Expression **(**Standard Bio Tools).
