# Supplementary material for: Impact of feeding diets without mineral P supplement on the immune system of two laying hen strains
Source: Poult Sci. 2026 Mar 13;105(6):106771. doi: 10.1016/j.psj.2026.106771 (PMC13052099; doi:10.1016/j.psj.2026.106771)
Supplement: Supplementary file 3 [file mmc3.pdf]

S3 – Supplementary Results

Table 1 Ingredients, calculated and analyzed compositions of experimental diets (already published in Sommerfeld et al., 2024)

	Developer		***Prelayer***		***Layer***	
Ingredients [g/kg]	*P-*	*P+*	*P-*	*P+*	*P-*	*P+*
Corn	619.5	617.1	589.8	585.8	599.2	595.9
Soybean meal	220.0	220.0	275.5	276.2	260.0	260.0
Alfalfa meal	120.0	120.0	60.0	60.0	30.0	30.0
Soybean oil	8.0	8.0	10.0	10.0	15.0	15.0
DL-Methionine	1.5	1.5	3.7	3.7	4.5	4.5
L-Lysine sulphate	-	-	-	-	0.7	0.7
Monocalcium phosphate	-	4.7	-	5.0	-	5.0
Limestone, fine	18.2	15.9	27.5	26.8	23.1	22.4
Limestone, coarse	-	-	20.0	19.0	54.0	53.0
Sodium chloride	2.3	2.3	2.8	2.8	3.0	3.0
Choline chloride	1.0	1.0	1.0	1.0	1.0	1.0
Sodium bicarbonate	2.0	2.0	2.2	2.2	2.0	2.0
Vitamin mix[1]	2.0	2.0	2.0	2.0	2.0	2.0
Mineral mix[2]	0.5	0.5	0.5	0.5	0.5	0.5
TiO_2	5.0	5.0	5.0	5.0	5.0	5.0
Calculated [g/kg]						
Phosphorus	3.0	4.0	3.3	4.3	3.1	4.1
Non phytate-phosphorus	1.3	2.3	1.4	2.4	1.3	2.3
Calcium	9.0	9.0	22.5	22.5	35.0	35.0
Crude protein	155	155	176	176	168	168
Metabolizable energy [MJ/kg]	11.6	11.6	11.5	11.5	11.5	11.5
Analyzed [g/kg dry matter]						
Phosphorus	-	-	-	-	3.6	4.9
Calcium	-	-	-	-	38.2	35.7
Crude protein	-	-	-	-	187	187
Myo-inositol	-	-	-	-	0.4	0.4
Ins(1,2,4,5,6)P_5	-	-	-	-	0.3	0.3
$InsP_6$	-	-	-	-	6.2	6.3
$InsP_6$-P	-	-	-	-	1.8	1.8
Non phytate-phosphorus	-	-	-	-	1.8	3.1
Ti	-	-	-	-	3.3	3.2

[1]Vitamin premix (Miavit GmbH, Essen, Germany), provided per kg of the complete diet: 10,000 IU vitamin A, 3,000 IU vitamin D3, 30 mg vitamin E, 2.4 mg vitamin K3, 100 mcg biotin, 1 mg folic acid, 3 mg vitamin B1, 6 mg vitamin B2, 6 mg vitamin B6, 30 mcg vitamin B12, 50 mg nicotinamide, 14 mg calcium-D-pantothenate.

[2]Trace element premix (Gelamin Gesellschaft für Tierernährung mbH, Memmingen, Germany), provided per kg of complete diet:80 mg manganese from manganese-(II)-oxide, 60 mg zinc from zinc sulfate monohydrate, 25 mg iron from ferrous-(II)-sulfate monohydrate, 7.5 mg copper from cupric-(II)-sulfate pentahydrate, 0.6 mg iodine from calcium iodate, 0.2 mg selenium from sodium selenite.

Table 2 Impact of dietary P, strain and period on immune cell frequencies in the blood, presented as proportions of immune cell subsets among total leukocytes [%] of laying hens

Dietary P	Strain	Monocytes	Heterophils	Total T Cells	Cytotoxic T Cells	$CD4^+$ T Cells	γδ T Cells	B Cells
P-	LB	5.29 ± 0.4	16.3 ± 1.2	48.7 ± 1.5	10.2 ± 0.5	24.3 ± 1.2	14.3 ± 0.8	6.81 ± 0.7
P-	LSL	4.55 ± 0.3	7.64 ± 0.5	49.7 ± 1.5	11.0 ± 0.5	20.4 ± 0.9	17.8 ± 0.8	9.78 ± 0.7
P+	LB	5.39 ± 0.4	17.9 ± 1.3	47.1 ± 1.5	9.81 ± 0.5	22.8 ± 1.1	14.0 ± 0.8	8.01 ± 0.7
P+	LSL	4.76 ± 0.3	8.06 ± 0.6	53.3 ± 1.5	11.4 ± 0.5	22.1 ± 1.0	19.3 ± 0.8	10.0 ± 0.7
**p-values**								
P × Strain × Period		0.171	0.373	0.255	0.920	0.382	0.093	0.208
P × Period		0.329	0.063	0.276	0.418	0.752	0.184	0.332
P × Strain		0.841	0.756	0.071	0.444	0.093	0.186	0.452
P		0.615	0.286	0.512	0.997	0.780	0.346	0.269
Strain		0.094	< 0.001	0.033	0.021	0.055	< 0.001	0.003
			LB > LSL	LB < LSL	LB < LSL		LB < LSL	LB < LSL

[1]Data are presented as LSmeans ± SEM.
[2]P- = without mineral P supplementation, P+ = supplemented with 1 g P / kg feed.
[3]LB = Lohmann Brown-Classic, LSL = Lohmann LSL-Classic.
[4]Results of statistical analysis of interactions (P × strain × period, P × period, P × strain) and main effects of P or strain using a linear mixed model are given. In case of significance (P < 0.05), significant differences of the post hoc test (Fisher LSD; P < 0.05) and direction of effects are stated below.

Table 3 Impact of dietary P, strain and period on the number of immune cells (×10^6) per g spleen of laying hens

Dietary P	Strain	Leukocytes	Macrophages	NK Cells	Total T Cells	Cytotoxic T Cells	CD4$^+$ T Cells	γδ T Cells	B Cells	CD4$^+$ CD25$^-$ T Cells	CD4$^+$ CD25^{dim} T Cells	CD4$^+$ CD25^{high} T Cells
P-	LB	1209 ± 61	5.14 ± 0.5	8.30 ± 0.7	854 ± 36	328 ± 19	216 ± 13	265 ± 14	301 ± 23	138 ± 10	37.3 ± 2.9	29.1 ± 2.4
P-	LSL	1576 ± 64	9.93 ± 1.1	12.1 ± 1.0	1172 ± 53	432 ± 26	251 ± 14	459 ± 26	308 ± 25	172 ± 13	43.9 ± 3.6	28.8 ± 2.5
P+	LB	1276 ± 62	5.45 ± 0 6	8.44 ± 0 7	887 ± 39	325 ± 19	226 ± 13	284 ± 16	350 ± 28	149 ± 11	38.8 ± 3.1	29.5 ± 2.4
P+	LSL	1688 ± 61	9.92 ± 1.1	12.7 ± 1.0	1259 ± 54	480 ± 28	270 ± 14	471 ± 25	351 ± 27	183 ± 14	46.1 ± 3.6	32.0 ± 2.4
**p-values**												
P × Strain × Period		0.322	0.448	0.669	0.267	0.937	0.204	0.244	0.345	0.232	0.082	0.324
P × Period		0.236	0.630	0.955	0.418	0.898	0.340	0.242	0.293	0.251	0.953	0.720
P × Strain		0.692	0.749	0.841	0.689	0.269	0.704	0.617	0.902	0.909	0.945	0.520
P		0.122	0.759	0.683	0.176	0.353	0.216	0.304	0.070	0.247	0.519	0.392
Strain		< 0.001	< 0.001	< 0.001	< 0.001	< 0.001	0.010	< 0.001	0.868	0.014	0.017	0.621
		LB < LSL	LB < LSL	LB < LSL	LB < LSL	LB < LSL	LB < LSL	LB < LSL		LB < LSL	LB < LSL	

[1]Data are presented as LSmeans ± SEM.

[2]P- = without mineral P supplementation, P+ = supplemented with 1 g P / kg feed.

[3]LB = Lohmann Brown-Classic, LSL = Lohmann LSL-Classic.

[4]Results of statistical analysis of interactions (P × strain × period, P × period, P × strain) and main effects of P or strain using a linear mixed model are given. In case of significance (P < 0.05), significant differences of the post hoc test (Fisher LSD; P < 0.05) and direction of effects are stated below

Table 4 Impact of dietary P, strain and period on immune cell frequencies in spleen, presented as proportions of immune cell subsets among total leukocytes [%] of laying hens

**Dietary P**	**Strain**	**Macrophages**	**NK Cells**	**Total T Cells**	**Cytotoxic T Cells**	**$CD4^+$ T Cells**	**γδ T Cells**	**B Cells**	**$CD4^+$ $CD25^-$ T Cells**	**$CD4^+$ $CD25^{dim}$ T Cells**	**$CD4^+$ $CD25^{high}$ T Cells**
P-	LB	0.44 ± 0.04	0.74 ± 0.05	72.8 ± 1.0	28.3 ± 1.0	17.5 ± 0.6	22.5 ± 1.1	26.4 ± 1.1	12.0 ± 0.7	3.28 ± 0.2	2.32 ± 0.2
P-	LSL	0.64 ± 0.07	0.85 ± 0.05	76.2 ± 1.1	28.2 ± 1.1	15.9 ± 0.6	29.7 ± 1.5	20.7 ± 1.1	11.3 ± 0.7	2.92 ± 0.2	1.77 ± 0.1
P+	LB	0.43 ± 0.04	0.68 ± 0.05	70.5 ± 1.0	26.1 ± 1.0	17.4 ± 0.6	22.6 ± 1.1	28.8 ± 1.1	12.1 ± 0.7	3.18 ± 0.2	2.23 ± 0.2
P+	LSL	0.60 ± 0.06	0.82 ± 0.05	75.8 ± 1.0	29.0 ± 1.0	15.9 ± 0.6	28.2 ± 1.4	21.9 ± 1.1	11.4 ± 0.7	2.87 ± 0.2	1.82 ± 0.1
**p-values**											
P × Strain × Period		0.142	0.295	0.767	0.219	0.301	0.846	0.412	0.550	0.202	0.853
P × Period		0.271	0.312	0.305	0.155	0.902	0.838	0.261	0.635	0.304	0.307
P × Strain		0.710	0.836	0.347	0.078	0.946	0.406	0.598	0.997	0.879	0.619
P		0.578	0.417	0.183	0.394	0.890	0.441	0.098	0.857	0.599	0.926
Strain		< 0.001	0.018	< 0.001	0.251	0.035	< 0.001	< 0.001	0.319	0.053	0.004
		LB < LSL	LB < LSL	LB < LSL		LB > LSL	LB < LSL	LB > LSL			LB > LSL

[1]Data are presented as LSmeans ± SEM.

[2]P- = without mineral P supplementation, P+ = supplemented with 1 g P / kg feed.

[3]LB = Lohmann Brown-Classic, LSL = Lohmann LSL-Classic.

[4]Results of statistical analysis of interactions (P × strain × period, P × period, P × strain) and main effects of P or strain using a linear mixed model are given. In case of significance (P < 0.05), significant differences of the post hoc test (Fisher LSD; P < 0.05) and direction of effects are stated below.

Table 5 Impact of dietary P, strain and period - summary of significant 3-fold interactions (P × strain × period) - on number and distribution of immune cells of laying hens

**Dietary P**	**Strain**	**Period**	**CD4^{+}CD25^{dim}** Cells /g cecal tonsils (× 10^{-6})	**NK cells** % of leukocytes in cecal tonsils
P-	LB	19 wk	1.68 ± 0.25	2.16 ± 0.21
P-	LB	24 wk	1.39 ± 0.21	2.76 ± 0.28
P-	LSL	19 wk	2.11 ± 0.31	1.72 ± 0.17
P-	LSL	24 wk	2.49 ± 0.37	1.07 ± 0.11
P+	LB	19 wk	1.26 ± 0.19	2.15 ± 0.22
P+	LB	24 wk	1.82 ± 0.27	2.02 ± 0.20
P+	LSL	19 wk	1.96 ± 0.29	1.16 ± 0.11
P+	LSL	24 wk	2.08 ± 0.31	1.02 ± 0.10
	**p-values**			
	P × Strain × Period		0.033	0.019
	**Effects**		LB P- 24 wk < LSL P- 24 wk<br>LB P+ 19 wk < LSL P+ 19 wk<br>LB P+ 19 wk < LB P+ 24 wk	LB P- 24 wk > LSL P- 24 wk<br>LB P- 24 wk > LB P+ 24 wk<br>LSL P- 19 wk > LSL P- 24 wk<br>LSL P- 19 wk > LSL P+ 19 wk<br>LB P+ 19 wk > LSL P+ 19 wk<br>LB P+ 24 wk > LSL P+ 24 wk

[1]Data are presented as LSmeans ± SEM.
[2]P- = without mineral P supplementation; P+ = supplemented with 1 g P / kg feed.
[3]LB = Lohmann Brown-Classic; LSL = Lohmann LSL-Classic.
[4]19 wk = before onset of egg laying; 24 wk = after onset of egg laying.
[5]Results of statistical analysis of interactions (P × strain × period) using a linear mixed model are given when P < 0.05. In case of interactions (P < 0.05), significant differences of the post hoc test (Fisher LSD; P < 0.05) and direction of effects are stated below.

Table 6 Impact of dietary P, strain and period on immune cell frequencies in the cecal tonsils, presented as proportions of immune cell subsets among total leukocytes [%] of laying hens

**Dietary P**	**Strain**	**NK Cells**	**Total T Cells**	**Cytotoxic T Cells**	**CD4⁺ T Cells**	**γδ T Cells**	**B Cells**	**CD4⁺CD25⁻ T Cells**	**CD4⁺CD25^dim T Cells**	**CD4⁺CD25^high T Cells**
P-	LB	2.44 ± 0.2	76.3 ± 1.6	35.1 ± 1.5	20.6 ± 1.2	15.5 ± 0.9	18.8 ± 1.6	12.9 ± 0.9	5.19 ± 0.4	2.38 ± 0.2
P-	LSL	1.36 ± 0.1	74.3 ± 1.6	27.2 ± 1.1	20.1 ± 1.1	22.2 ± 1.3	23.4 ± 1.5	11.1 ± 0.8	6.16 ± 0.4	2.70 ± 0.2
P+	LB	2.08 ± 0.2	76.7 ± 1.7	38.4 ± 1.6	17.3 ± 1.0	15.5 ± 0.9	18.8 ± 1.6	10.8 ± 0.8	4.83 ± 0.4	1.77 ± 0.1
P+	LSL	1.09 ± 0.1	73.3 ± 1.5	27.1 ± 1.1	20.5 ± 1.2	20.8 ± 1.2	24.2 ± 1.5	12.1 ± 0.9	5.58 ± 0.4	2.65 ± 0.2
**p-values**										
P × Strain × Period		0.019	0.298	0.154	0.324	0.480	0.128	0.613	0.154	0.477
P × Period		0.895	0.375	0.137	0.914	0.941	0.504	0.895	0.643	0.539
P × Strain		0.621	0.633	0.198	0.053	0.560	0.791	0.057	0.761	0.053
P		0.007<br>P- > P+	0.835	0.234	0.136	0.589	0.796	0.521	0.182	0.028<br>P- > P+
Strain		< 0.001<br>LB > LSL	0.119	< 0.001<br>LB > LSL	0.291	< 0.001<br>LB < LSL	0.008<br>LB < LSL	0.826	0.051	0.003<br>LB < LSL

[1]Data are presented as LSmeans ± SEM.
[2]P- = without mineral P supplementation, P+ = supplemented with 1 g P / kg feed.
[3]LB = Lohmann Brown-Classic, LSL = Lohmann LSL-Classic.
[4]Results of statistical analysis of interactions (P × strain × period, P × period, P × strain) and main effects of P or strain using a linear mixed model are given. In case of significance (P < 0.05), significant differences of the post hoc test (Fisher LSD; P < 0.05) and direction of effects are stated below.

Table 7 Impact of dietary P, strain and period on antibody concentration in plasma and bile of laying hens

Dietary P	Strain	Plasma IgY [mg/ml]	Plasma IgM [µg/ml]	Plasma IgA [µg/ml]	Bile IgA [mg/ml]
P-	LB	9.65 ± 0.6	403 ± 23	305 ± 17	42.6 ± 5.3
P-	LSL	9.64 ± 0.6	405 ± 23	181 ± 17	34.0 ± 4.3
P+	LB	9.39 ± 0.6	381 ± 22	315 ± 17	33.7 ± 4.1
P+	LSL	9.47 ± 0.6	428 ± 25	187 ± 17	35.2 ± 4.2
**p-values**					
P × Strain × Period		0.962	0.321	0.997	0.551
P × Period		0.709	0.182	0.536	0.052
P × Strain		0.936	0.243	0.845	0.272
P		0.687	0.976	0.507	0.410
Strain		0.957	0.370	< 0.001<br>LB > LSL	0.464

[1]Data are presented as LSmeans ± SEM.
[2]P- = without mineral P supplementation, P+ = supplemented with 1 g P / kg feed.
[3]LB = Lohmann Brown-Classic, LSL = Lohmann LSL-Classic.
[4]Results of statistical analysis of interactions (P × strain × period, P × period, P × strain) and main effects of P or strain using a linear mixed model are given. In case of significance ($P < 0.05$), significant differences of the post hoc test (Fisher LSD; $P < 0.05$) and direction of effects are stated below.

Table 8 Impact of dietary P, strain and period on gene expression of pro- and anti-inflammatory cytokines, foxp3 and genes related to oxidative stress reactions in the spleen of laying hens

Dietary P	Strain	IL-1β	IL-2	IL-6	IL-12	TNF-α	IFN-γ	SOD1	SOD2	iNos	Foxp3	IL-10	IL-17
					[$2^{-\Delta Cq}$ × 10^{-4}][1]							[%/100]	
P-	LB	7.5 ± 1.0	13.9 ± 1.4	1.3 ± 0.3	3.9 ± 0.6	17.9 ± 1.1	3.5 ± 0.6	1674 ± 60	617 ± 26	178 ± 13	437 ± 37	0.34 ± 0.1	0.09 ± 0.1
P-	LSL	21.7 ± 2.7	13.3 ± 1.4	1.2 ± 0.2	3.3 ± 0.5	24.5 ± 1.5	1.9 ± 0.3	1719 ± 62	561 ± 24	235 ± 17	496 ± 42	0.39 ± 0.1	0.10 ± 0.1
P+	LB	6.2 ± 0.8	11.0 ± 1.2	1.6 ± 0.3	3.9 ± 0.7	15.9 ± 1.0	2.8 ± 0.5	1648 ± 61	577 ± 25	157 ± 12	427 ± 37	0.19 ± 0.1	0.13 ± 0.1
P+	LSL	20.1 ± 2.5	12.8 ± 1.3	0.9 ± 0.2	4.4 ± 0.7	26.2 ± 1.6	1.7 ± 0.3	1743 ± 62	540 ± 23	238 ± 17	495 ± 42	0.44 ± 0.1	0.24 ± 0.1
**p-values**													
P × Strain × Period		0.624	0.589	0.461	0.036	0.362	0.191	0.462	0.722	0.844	0.857	-	-
P × Period		0.949	0.429	0.309	0.822	0.962	0.410	0.566	0.871	0.579	0.659	0.350	0.124
P × Strain		0.650	0.340	0.227	0.417	0.137	0.642	0.646	0.739	0.278	0.886	0.337	0.656
P		0.279	0.210	0.701	0.349	0.672	0.248	0.974	0.211	0.363	0.868	0.586	0.331
Strain		< 0.001	0.636	0.162	0.887	< 0.001	0.003	0.269	0.078	< 0.001	0.166	0.278	0.551
		LB < LSL				LB < LSL	LB > LSL			LB < LSL			

[1]Data are presented as $2^{-\Delta Cq}$-transformed LSmeans ± SEM of ΔCq values, with ΔCq = mean Cq $_{\text{(gen of interest)}}$ – mean Cq $_{\text{(reference genes)}}$.

[2]For IL-10 and IL-17, relative proportion [%] of expression frequency is given (n=80).

[3]P- = without mineral P supplementation, P+ = supplemented with 1 g P / kg feed.

[4]LB = Lohmann Brown-Classic, LSL = Lohmann LSL-Classic.

[5]Results of statistical analysis of interactions (P × strain × period, P × period, P × strain) and main effects of P or strain using a linear mixed model or generalised linear model are given. In case of significance (P < 0.05), significant differences of the post hoc test (Fisher LSD; P < 0.05) and direction of effects are stated below.

S3 – Supplementary Results

Table 9 Impact of dietary P and period – summary on significant 2-fold interactions (P × period) - on gene expression of pro- and anti-inflammatory cytokines in the cecal tonsils of laying hens

Dietary P	Period	IL-2	IL-6	IL-10	IL-12
			$[2^{-\Delta Cq} \times 10^{-4}]^{1}$		
P-	19 wk	10.9 ± 0.62^{ab}	0.15 ± 0.02^{ab}	0.73 ± 0.08^{ab}	0.56 ± 0.05^{a}
P-	24 wk	12.0 ± 0.68^{a}	0.18 ± 0.03^{ab}	0.86 ± 0.10^{a}	0.43 ± 0.04^{ab}
P+	19 wk	12.1 ± 0.69^{a}	0.19 ± 0.03^{a}	0.91 ± 0.10^{a}	0.42 ± 0.04^{b}
P+	24 wk	9.97 ± 0.57^{b}	0.13 ± 0.02^{b}	0.62 ± 0.07^{b}	0.51 ± 0.05^{ab}
**p-values**					
P x Period		0.007	0.025	0.016	0.020

[1]Data are presented as $2^{-\Delta Cq}$-transformed LSmeans ± SEM of ΔCq values, with ΔCq = mean $Cq_{\text{(gen of interest)}}$ – mean $Cq_{\text{(reference genes)}}$.
[2]P- = without mineral P supplementation, P+ = supplemented with 1 g P / kg feed.
[3]19 wk = before onset of egg laying; 24 wk = after onset of egg laying.
[4]Results of statistical analysis of interactions (P × period) using a linear mixed model are given when P < 0.05. In the case of interactions (P < 0.05), different superscript letters indicate differences in means (Fisher LSD; P < 0.05).
